# The Multifaceted Nature of Macrophages in Cardiovascular Disease

**DOI:** 10.3390/biomedicines12061317

**Published:** 2024-06-13

**Authors:** Cindy X. Li, Lixia Yue

**Affiliations:** 1 Department of Cell Biology, Pat and Jim Calhoun Cardiovascular Center, University of Connecticut Health Center, Farmington, CT 06030, USA; cindli@uchc.edu; 2 Institute for the Brain and Cognitive Sciences, University of Connecticut, Storrs, CT 06269, USA

**Keywords:** macrophages, cardiovascular disease, macrophage-mediated inflammation, atherosclerosis, lipid metabolism

## Abstract

As the leading cause of mortality worldwide, cardiovascular disease (CVD) represents a variety of heart diseases and vascular disorders, including atherosclerosis, aneurysm, ischemic injury in the heart and brain, arrythmias, and heart failure. Macrophages, a diverse population of immune cells that can promote or suppress inflammation, have been increasingly recognized as a key regulator in various processes in both healthy and disease states. In healthy conditions, these cells promote the proper clearance of cellular debris, dead and dying cells, and provide a strong innate immune barrier to foreign pathogens. However, macrophages can play a detrimental role in the progression of disease as well, particularly those inflammatory in nature. This review will focus on the current knowledge regarding the role of macrophages in cardiovascular diseases.

## 1. Introduction

Despite enormous advances in therapy development, cardiovascular disease (CVD) remains the leading cause of mortality worldwide [1,2]. The prevalence of CVD (including coronary heart disease, heart failure, stroke, and hypertension) in adults 20 years or older is approximately 50% (approximately 130 million reported cases), which increases with age in both males and females [3]. Based on the most recent reports, CVDs cause approximately 20 million deaths globally each year, with heart disease and stroke claiming more lives than cancer and lower respiratory disease combined [1]. Overwhelmingly, the field of cardiovascular research has defined a distinct role for the immune system in mediating pathological CVD progression and/or resolution and regression [4].

Macrophages are a highly diverse immune cell type that are found ubiquitously in tissues with varying localizations and specificities. They are responsible for an array of processes, ranging from the development of organ systems to injury response [5,6,7,8]. The sub-classification of macrophages has been widely explored with single-cell sequencing techniques with a variety of disease backgrounds [9,10,11,12,13]. However, the disparity in the classifications of the different subtypes and varied use of macrophage cellular and polarization markers in the literature poses a non-uniform approach regarding macrophage research and therapeutic targeting. The role of macrophages in cardiovascular disease has gained increasing attention due to the rising prevalence of CVD-related deaths globally. Recent research has placed particular emphasis on the inflammatory and immunological backgrounds of CVDs as an area with high therapeutic potential.

**Figure 1:**
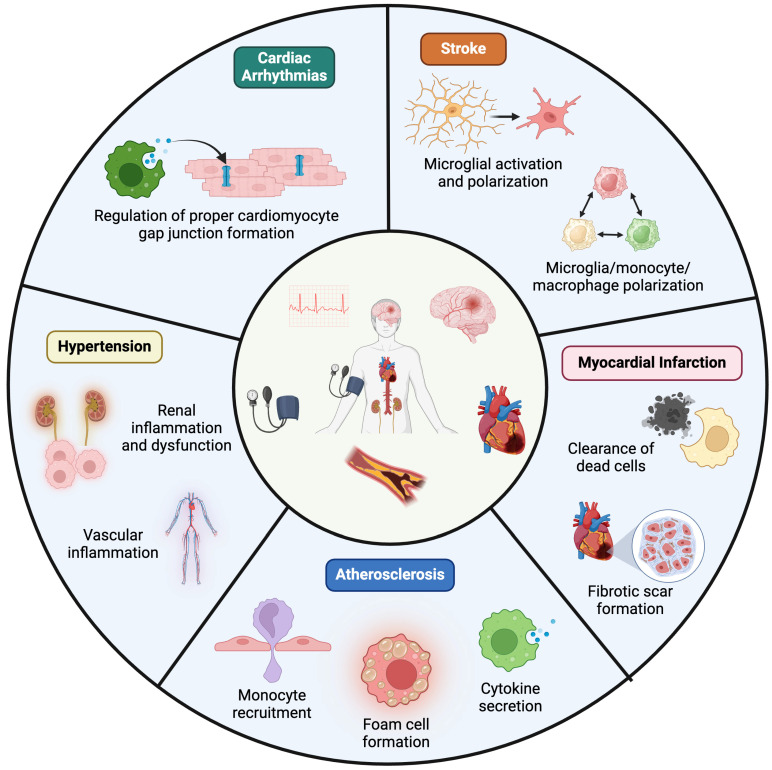
**Overview of Key Macrophage Functions in Cardiovascular Diseases.** Cardiovascular disease encompasses a range of conditions, including stroke, myocardial infarction, atherosclerosis, hypertension, and arrythmias such as atrial fibrillation. This schematic diagram illustrates the roles and functions of the immune system, specifically focusing on macrophages, in these diseases.

The purpose of this review is to highlight macrophage diversity and their contributions to various CVDs, including atherosclerosis and myocardial infarction (Figure 1). The dysregulation of several key macrophage-mediated processes, including macrophage polarization, cytokine secretion, and macrophage-mediated efferocytosis, is shared among many CVDs as well as other inflammatory diseases. A better understanding of these processes will enable the development of more effective therapies for patients.

## 2. Regulation of Macrophage Diversity and Function in Healthy and Disease States

Macrophages are a highly diverse cell type and possess a range of phenotypes and diverse functions. Generally, macrophages are important regulators of nearly all inflammatory processes, in which they can promote or repress inflammation by clearing dying cells and cellular debris, and secreting chemokines and cytokines that can lead to increased immune cell recruitment. Additionally, macrophages are important regulators of tissue development, tumor growth, and wound repair. Macrophages have been termed as “M0-like” (non-polarized, “naive” state [14]), “M1-like” (inflammatory or “classically activated”), or “M2-like” (alternatively activated), but the increased sophistication of macrophage genotyping and phenotyping has shown that macrophages exist in a fluid continuum [15,16,17]. Macrophage plasticity is essential for their ability to adopt different characteristics based on the tissue location, inflammatory environment, and presence of injury-related cytokines and signaling factors. However, this phenotype plasticity causes macrophages to be a complex cell type to study due to their heterogeneity.

Macrophage polarization towards M1- or M2-like states was first explored in the context of microbial and foreign pathogen challenge and injury, but studies have confirmed a role for host states in determining the response of these innate immune cells. The soluble cytokine interferon-gamma (IFN*γ*) was the first identified macrophage-activating factor and induces M1 polarization. Purified IFN*γ* increased hydrogen peroxide production in human macrophages as a way to kill intracellular pathogens such as *T. gondii* [18]. The identification of the transcription factors responsible for promoting macrophage plasticity or polarization, such as NFκB and KLF4 [19], have revealed complex mechanisms by which macrophages adjust their features to accommodate healthy or diseased states. Later, it was shown that type I interferons largely exert their transcriptional regulation by modulating pathways like JAK/STAT [20]. In addition to interferons, toll-like receptors (TLRs) can activate a canonical IRF-STAT signaling pathway for M1 polarization, primarily modulated by STAT1 [21]. In contrast, anti-inflammatory interleukins like IL-4 and IL-13 can trigger an M2-like phenotype through STAT6 [21]. STAT3/5 are typically seen to be involved in M2 polarization as well [22,23].

Attempts at characterizing markers to differentiate between M1- and M2-like macrophages have been challenging due to the characterization method used or the disease or genetic background of the animal model. Nonetheless, some of the most widely accepted markers for pro-inflammatory, M1-like macrophages include inducible nitric oxide synthase (iNOS), interleukin-beta (IL-1β), tumor necrosis factor-alpha (TNFɑ), IL-12, and more [24,25]. Additional markers include CD38, G-protein-coupled receptor 18 (Gpr18), and formyl peptide receptor 2 (Fpr2) [26]. Conversely, the markers for anti-inflammatory, M2-like macrophages include arginase 1 (Arg1) and CD206, two of the most commonly used M2-like macrophage markers. Early growth response protein 2 (Egr2) and c-Myc have also been suggested to be M2-exclusive [26]. These markers have been widely used to identify macrophage identities in experiments including flow cytometry and immunostaining. The concept of M1- and M2-like macrophages, their plasticity, and their classification have been the topic of many other in-depth reviews and critiques and will not be explored in detail here [26,27,28,29,30,31,32,33]. In this review, macrophages will be referenced as either “M1-like” or “M2-like”, as these are useful terms to distinguish between macrophages with generally opposing phenotypes.

## 3. Macrophages in Cardiovascular Disease

### 3.1. Macrophages in Atherosclerosis

Atherosclerosis, a disease that is characterized by an accumulation of fatty lesions of major arteries, is the underlying cause of 50% of deaths in Western societies. In total, 75% of acute myocardial infarctions occur from the rupture of the luminal plaques. Additionally, ischemic stroke, the major form of stroke, is due to atherosclerotic cardiovascular disease [34,35]. Current treatments for atherosclerosis include statins, which aim to control the disease by targeting lipid metabolism and reducing cholesterol levels in the blood. In severe cases, surgical interventions may be employed to manually remove plaque from the inside of the arteries. However, the identification of atherosclerosis as a chronic inflammatory disease characterized by increased immune cell infiltration and recruitment to plaques has paved the way for inflammation-targeted research and treatment options for this disease. Macrophages comprise the majority of cells within the fatty plaques and are major drivers for not only the initiation of the disease, but also the positive feedback loop responsible for increased tissue inflammation in plaque tissue. Given the crucial role of macrophages in atherogenesis, it is unsurprising that research on macrophages in atherosclerosis is among the most extensively studied.

#### 3.1.1. Increased Macrophage Accumulation in the Plaque Increases Atherosclerosis

**Figure 2:**
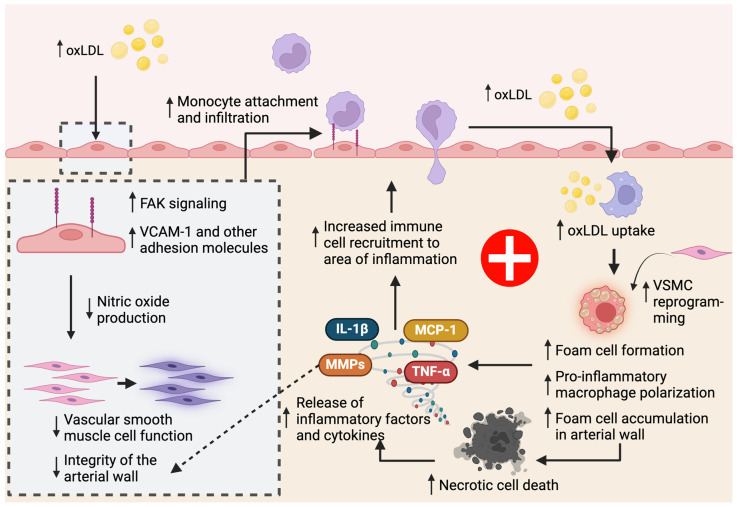
**Macrophage Regulation of Inflammatory Processes in Atherosclerosis.** Levels of serum oxLDL are elevated in atherosclerosis, either due to environmental factors such as a fatty diet or dysregulated lipid metabolism. oxLDL can promote the activation of endothelial cells and promote focal adhesion kinase (FAK) signaling pathways that upregulate the expression of adhesion molecules such as VCAM-1 in endothelial cells. oxLDL has also been shown to affect normal nitric oxide production in endothelial cells, which greatly affects the proper functioning of underlying smooth muscle cells and compromises vascular integrity. Recruited macrophages are highly implicated in the progression of the atherosclerotic plaque. They are responsible for the metabolism and clearance of oxLDL and the induction of VSMC reprogramming, they undergo macrophage polarization towards M1- or M2-like macrophage phenotypes and release cytokines and chemoattractant proteins to facilitate increased leukocyte invasion, and their non-apoptotic cell death within the plaque causes plaque instability and increased inflammation.

A major risk factor and initiator for atherosclerosis is low-density lipoprotein (LDL). Unlike high-density lipoprotein (HDL), which promotes proper lipid metabolism and clearance from the body, LDL is a non-beneficial form of lipoprotein that contributes to the buildup of atherosclerotic plaques. Generally, LDL can be modified into different forms of LDL that are often associated with disease, including oxidized [36,37], acetylated [38], ethylated [39], methylated [39], and glycated LDL [40]. In atherosclerosis, oxidized LDL (oxLDL) levels are elevated in the bloodstream, where it can increase endothelial NF*κ*B activation. oxLDL will also affect endothelial cell function, hindering the normal nitric oxide production of endothelial cells, which in turn affects underlying cells like vascular smooth muscle cells [41], leading to compromises in the integrity of the arterial wall. In the early stages of atherosclerosis, M1-like (Ly6-C^high^) monocytes in the bloodstream will encounter activated endothelial cells that have responded to cellular stressors, such as oxLDL, by upregulating adhesion molecules, which will promote the recruitment of immune cells to the area to relieve inflammation [42,43,44,45]. Mechanistically, oxLDL increases endothelial focal adhesion kinase (FAK) signaling, leading to increased VCAM-1 expression, and pro-inflammatory adhesion molecule upregulation [46] (Figure 2).

The recruitment of monocytes into the area is initially aimed at metabolizing accumulated oxLDL, but the trapping and buildup of lipid-laden macrophages (“foam cells”) within the arterial walls will ultimately cause atherosclerosis and promote a cycle of increasing oxidative stress in surrounding tissues. oxLDL has also been shown to be a selective inducer of human monocyte activation, stimulating infiltrated monocytes, which differentiate into macrophages, to release cytokines such as monocyte chemoattractant protein 1 (MCP-1) and CXCL1. The positive feedback loop of monocyte recruitment, infiltration, and differentiation into macrophages to and within the inflamed vascular tissues is the initiating trigger for macrophage accumulation and plaque buildup [47,48]. Interestingly, lesional macrophage accumulation within the atheroma has been attributed to macrophage proliferation, a process unaffected by intracellular lipid accumulation, rather than increased monocyte recruitment [49]. oxLDL has also been shown to induce the macrophage-mediated production of pro-thrombotic particles, which compromises the stability of the plaque [50]. Within the plaque, M1-like macrophages have been suggested to promote unstable, increased plaques, while the enrichment of M2-like macrophages, which are mediated by chemokine receptors such as CCR2 and CX3CR1, has been linked to plaque regression in vivo [51,52,53]. Metformin, one of the most highly prescribed drugs for a variety of diseases, has been suggested to regulate and promote M2 macrophage polarization in obesity and atherosclerosis by activating M2 signaling pathways like AMPK and inhibiting NF*κ*B activation pathways [54,55,56,57,58].

#### 3.1.2. Dysfunctional Macrophage Lipoprotein Metabolism Contributes to Atherosclerosis

Recognizing the detrimental effects of improper oxLDL lipid metabolism on the initiation of atherogenesis, targeting lipid uptake or efflux in atherosclerotic macrophage metabolism appears to be a promising approach for attenuating atherosclerosis. Genome-wide screening can identify target genes and proteins involved in the promotion of macrophage foam cell formation and dysregulated lipid uptake. For example, a genome-wide CRISPR screen conducted by Patterson et al. identified Trem2, a lipid sensor that promotes oxLDL uptake by foam cells, in the promotion of atherosclerosis. The subsequent myeloid-specific deletion of Trem2 attenuated atherosclerosis in vivo [59]. In a combined rheumatoid arthritis–atherosclerosis model, Walker et al. showed that the modulation of the lipid metabolism process using resolvin T4, a lipid metabolite that functions to alleviate inflammation [60], induced increased cholesterol efflux from lipid-laden macrophages, thereby attenuating atherosclerosis [61]. Other mediators that are involved in the metabolic programming of atherosclerotic macrophages include fatty acids, scavenger receptors, glucose, ROS, oxygen levels [62], sorting nexin 10 [63], lysophosphatidylglucoside/GPR55 in human macrophages [64]. Macrophages can disrupt the initiation of atherosclerosis by mediating the appropriate uptake and metabolism of lipids. Conversely, atherogenesis is triggered when this macrophage-mediated process is dysregulated.

#### 3.1.3. Macrophages Promote Inflammatory Cell Death Pathways in Atherosclerosis

A major mechanism by which macrophages promote atherosclerosis is by exacerbating the inflammatory plaque environment. The initial response to elevated levels of oxLDL is the macrophage-mediated uptake and metabolism of these lipids, which promote “foam cell” formation. When foam cells become trapped within the arterial walls and plaque lesions, high levels of cellular stress are triggered within these macrophages. The disruption in homeostasis of these foam cells can lead to a deviation away from proper lipid clearance phenotypes and towards pro-inflammatory macrophage phenotypes (Figure 2). Within the atherosclerotic plaque, Li et al. have identified populations of homeostatic and inflammatory foam cells using a novel computational program AtheroSpectrum [65]. Such cellular stress results in elevated levels of inflammatory cell death within the plaque environment, which will not only increase plaque instability but also promote inflammation via cytokine production and immune cell recruitment in a preliminary attempt to resolve tissue inflammation. Two categories of inflammatory cell death are necroptosis and pyroptosis [66,67], both of which have been shown to be distinct pathways that contribute to atherosclerotic inflammation.

In macrophages, oxLDL has been shown to promote necroptotic cell death by directly activating and upregulating the expression of necroptotic genes RIP3 and MLKL [66]. As a result of extracellular stressors such as oxLDL, oxidized phospholipids, lipoproteins, and saturated fatty acids, intracellular reactive oxygen species (ROS) and ER stress can also result in cell death [68]. Recently, the involvement of the NLRP3 inflammasome in inflammatory diseases has gained traction as a possible target for the reduction of inflammatory cytokine production and tissue inflammation [69,70]. Both oxLDL and LDL can serve as priming factors for the upregulation of NLRP3 inflammasome expression. Other priming signals for NLRP3 expression in inflammation include pro-inflammatory gene transcription factor NFκB [71]. Other triggers for NLRP3 inflammasome activation have also been suggested, including decreased cellular cAMP and post-translational modifications [72,73]. Although various stimuli have been identified to trigger NLRP3-mediated inflammation, the exact mechanisms by which NLRP3 is activated are yet to be fully elucidated. Recently, Orecchioni et al. discovered that olfactory receptor 2 (Olfr2), a protein that was considered to be largely uninvolved in the regulation of inflammatory signaling, is a novel regulator of macrophage-NLRP3-mediated IL-1 cytokine production and inflammation in atherosclerosis [74]. The novel properties of upstream NLRP3 priming and activation signals highlight a need to further elucidate the regulation of NLRP3 in order to better understand macrophage NLRP3 as a possible target for curbing cell death and the ensuing inflammatory responses in CVDs.

#### 3.1.4. Efferocytotic Macrophages Are Atheroprotective

One of the major roles of macrophages is efferocytosis, or the specific phagocytic clearance of dead and dying cells. In the early stages of atherogenesis, macrophages play a critical role in the clearance of foam cells and the proper metabolism of oxLDL. oxLDL has also been shown to damage the lysosomal membranes of cells, thereby affecting the phagocytic and efferocytotic ability of macrophages [75]. Recognizing the importance of such a process, potential therapies to reactivate or upregulate the ability of plaque macrophages to polarize towards a pro-phagocytic and efferocytotic phenotype may lead to the better management or reversal of atherosclerotic symptoms. Interestingly, diet-derived molecules such as protocatechuic acid, which is enriched in a polyphenol-rich diet including certain fruits and vegetables, have been shown to increase continual macrophage efferocytosis through the upregulation of major efferocytotic receptor MER tyrosine receptor (MerTK), which increases the clearance of dying cells in the atheroma and reduces the size of necrotic cores in plaques in vivo [76]. In the case of protocatechuic acid, intermediate factors in the MerTK upregulation signaling cascade such as microRNA (miR10-b), which have known inhibitors or activators, may also serve as more feasible therapeutic targets to modulate the macrophage phenotype [76]. This is an interesting example of how diet may have an atheroprotective effect.

#### 3.1.5. Ion Channels Modulate Macrophage Function in Atherosclerosis

Intracellular second messenger molecules, particularly ions such as calcium and potassium, play a critical role in modulating extracellular stressors such as oxLDL into a cellular response. For example, calcium-mediating signaling can also trigger NLRP3 activation via G-protein-coupled Ca^2+^ sensors or extracellular calcium-sensing receptors (CASRs) [77,78,79], among other mechanisms. In inflammation, necrotic cells are a source of extracellular calcium, increasing the ion’s concentration in inflamed tissues. Rising intracellular calcium, such as that mediated by phosphatidyl inositol or mitochondrial damage or ER-stress-mediated Ca^2+^ release, can activate the assembly of the inflammasome and caspase-1 activation, thereby releasing inflammatory cytokines such as interleukins into the extracellular environment [77,80] and promoting disease-associated inflammation. This emphasizes the importance of ion channel regulation in macrophages and presents several predominant ion channel types, including potassium channels, transient receptor potential (TRP) channels, calcium channels, mechanosensitive Piezo channels, chloride channels, and proton channels, as potential therapeutic targets for modulating macrophage phenotype and function [81,82].

In atherosclerosis, the most commonly studied channels are the Piezo and transient receptor potential (TRP) ion channels, the latter being a family of channels that serve as cellular sensors of a variety of stimuli such as stress, temperature, and stretch. There are 28 mammalian TRP channels that are sub-classified into six subfamilies based on amino acid sequence homology: TRPA (ankyrin), TRPC (canonical), TRPM (melastatin), TRPV (vanilloid), TRPML (mucolipin), and TRPP (polycystin). TRP ion channels are found in a variety of tissues, with some having more tissue specificity than others. TRP channels are permeable to a wide range of ions including, but not limited to, Ca^2+^, Mg^2+^, Zn^2+^, Na^+^, K^+^, and more [83,84]. The atomic structures of many of the TRP channels have been elucidated, and their unique structures make them attractive, specific therapeutic targets for various diseases. Multiple TRP channels (e.g., TRPA1, TRPV1, TRPV4, TRPC3, TRPM2, and TRPM7 [85,86,87,88,89,90,91,92,93,94,95,96,97]) have been suggested to regulate macrophages.

However, despite in vitro results showing the TRP channel-mediated modulation of macrophage phenotypes, only a few TRP channels have been shown to affect in vivo atherosclerotic phenotypes. These channels play a crucial role in the promotion or inhibition of atherosclerosis in a cell-type-specific manner, making this class of channels interesting to explore in the context of channel-mediated signaling in inflammation and disease. For example, TRPV1 activation led to reduced lipid storage and atherosclerotic lesions in a high-fat diet Apoe^−/−^ model [98]. TRPA1 has also been implicated in foam cell formation by altering cholesterol efflux in vivo [99]. Moreover, TRPV4 has been shown to increase monocyte adhesion and thus atherosclerotic initiation in vivo [100]. The transplantation of TRPC3^−/−^ bone marrow into wild-type mice fed a high-fat diet for three weeks resulted in smaller plaques and other atherosclerotic phenotypes such as necrotic core size compared to controls in vivo [101,102]. miR-26a was also found to evoke anti-atherosclerotic phenotypes by targeting TRPC3 and reducing the associated inflammatory responses [103]. TRPM2, a Ca^2+^-permeable channel that is activated by oxidative stress, has been shown to contribute to atherogenesis in a macrophage-specific manner. TRPM2 has been suggested to modulate the excessive ROS production associated with inflammation into a Ca^2+^-mediated inflammatory signaling pathway, such as that mediated by the NLRP3 inflammasome [104,105]. TRPM2 can also contribute to atherosclerotic progression at the level of macrophage foam cell formation. Zong et al. found that global and macrophage-specific TRPM2 deletion attenuated atherosclerosis. Mechanistically, they found that this effect was due to the reduction of oxLDL uptake and foam cell formation and that TRPM2 was an important regulator of the macrophage CD36 pathway, which is well known to be critical in the uptake of oxLDL in atherosclerosis [106]. TRP channels and their unique conductances to certain ions, particularly calcium, and their activation by a variety of atherosclerosis-associated stimuli (i.e., oxidative stress) in specific cell types underscore the importance of TRP channels in macrophages in atherogenesis. Thus, the role of ion channels, particularly those conducive to calcium and necessary for regulating ion homeostasis within the cell, is indisputably important for identifying pro-inflammatory signaling mechanisms.

#### 3.1.6. Heterogeneity of Macrophages in the Atherosclerotic Plaque

Interestingly, recent studies have suggested that the macrophage population within the atherosclerotic plaque may not be as homogeneous as believed. For example, atherosclerotic plaque macrophages within the atherosclerotic plaque have long been believed to be either of monocyte or resident macrophage origin. However, single-cell sequencing experiments have shown that many or most of the macrophages within the atherosclerotic plaque are of vascular smooth muscle (VSMC) origin. Aortic VSMCs can undergo a process termed VSMC “reprogramming” or “phenotype switching” [107,108]. The factors that promote this VSMC de- and redifferentiation into a macrophage-like state have yet to be fully elucidated. Additionally, elevated macrophage plasticity in response to the aberrant inflammatory plaque environment can also alter the balance between pro- and anti-inflammatory macrophage subtypes. For example, certain signaling pathways, including a lysophosphatidylglucoside/GPR55 regulatory pathway, were shown to promote a pro-foam cell phenotype in human M2-like macrophages, which are typically characterized as resolvers and suppressors of innate inflammation [64]. The heterogeneity of macrophages within the atherosclerotic plaque has slowly been better understood using different genetic profiling techniques featuring distinct cellular markers or transcriptional or behavioral profiles. Using scRNA-seq on IFNγ-stimulated human macrophages derived from peripheral mononuclear cells, Decano et al. identified two distinct clusters of IFNγ-activated macrophages in vitro: those that were inflammatory and those that were phagocytic macrophages. Each of these macrophages had distinctive chemotactic, secretory, and metabolic profiles. An analysis of human carotid artery plaque samples recapitulated this finding [109].

#### 3.1.7. Understanding Macrophages as Potential Targets for Atherosclerosis

In addition to elucidating the populations and roles of macrophage types in atherosclerosis, the targeting of macrophages, particularly those that exacerbate inflammation in the tissue microenvironment, has shown success in in vitro and in vivo models of atherosclerosis. The suppression of inflammatory genes using the compound BI-2536 in human macrophages in vitro decreased the overall ratio of IFNγ-activated inflammatory macrophages and resulted in a significant reduction in atherosclerotic burden in vivo using the Ldlr^−/−^ atherosclerotic mouse model [109]. The use of macrophage-targeting nanoparticles to regulate the expression of certain transcription factors, such as ZEB1, that can regulate macrophage pro- or anti-inflammatory phenotype also represents a treatment delivery method in vivo [110]. A better understanding of the specific modulatory processes in macrophages in atherosclerosis is necessary to not only disrupt atherosclerotic progression in an effective manner, but also to avoid any undesirable, off-target effects of macrophage-targeted treatments. The prevalence of well-established atherosclerosis murine models, such as the Apoe^−/−^ hypercholesteremia model or the Ldlr^−/−^ model, establishes an excellent foundation for continued research into the above-mentioned pro-atherogenesis processes and will provide a basis for branched research into the role of macrophages in hypercholesteremia/atherosclerosis comorbidities such as stroke and myocardial infarction.

### 3.2. Macrophages in Myocardial Infarction

Acute coronary syndrome is defined as one instance or an “acute” manifestation of coronary heart disease. From an epidemiology and public health perspective, acute coronary syndrome is a massive problem, affecting over one million patients per year and costing approximately $8 billion US dollars on the care and management of patients post-injury [1]. Acute coronary syndrome is typically associated with the loss of proper oxygenation in the heart tissue (myocardial ischemia), and myocardial infarction (MI), an acute coronary disease, is the single highest cause of heart failure and cardiac-associated morbidity and mortality [111,112,113,114]. The estimated annual occurrence of MI is 605,000 new attacks and 200,000 recurrent attacks [1]. MI is an acute coronary syndrome that is caused by vascular occlusion(s) in the cardiac vasculature, which deprives areas of the myocardium of oxygen (ischemia) and nutrients, leading to widespread tissue damage and cell death in the infarcted tissue. Such occlusions are typically caused by thrombosis or by the rupture of an unstable atherosclerotic plaque [115,116].

**Figure 3:**
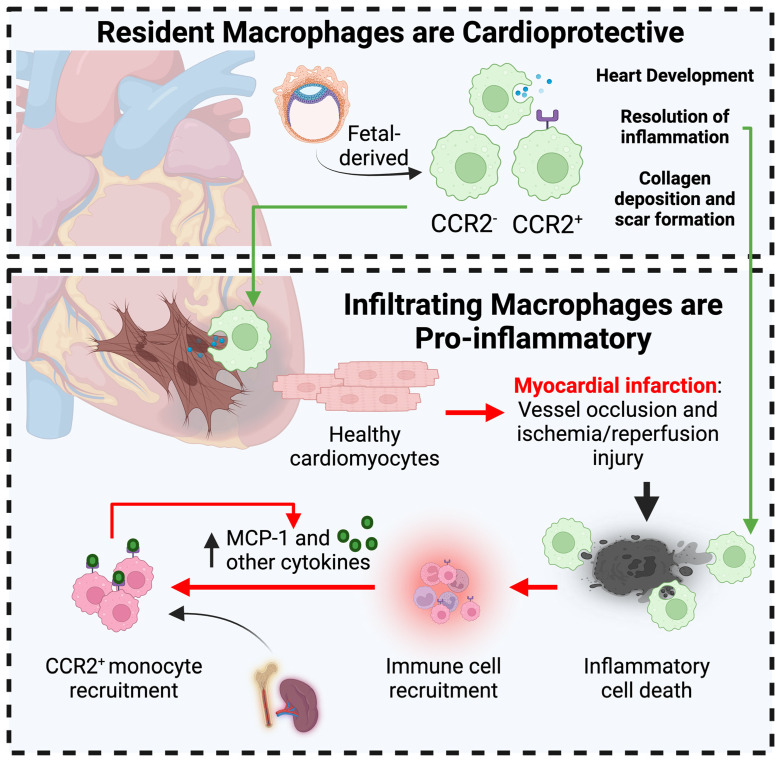
**Divergent roles of macrophages in the healthy and post-MI heart.** Resident macrophages are generally thought to be cardioprotective cells that exhibit an M2-like phenotype and markers. They are fetal-derived macrophage populations that are typically defined as CCR2^+^ or CCR2^−^ depending on their origins. In MI, these resident cardiac macrophages play protective roles including efferocytosis of cell debris and dead cells and promoting scar formation in the infarct. Conversely, infiltrated macrophage populations post-MI contribute to increased inflammation and immune cell recruitment, which can alter the microenvironment of the infarcted tissue, and dysfunctional inflammation resolution can result in chronic loss of cardiac function.

Therapies for MI are targeted at saving the infarcted, ischemic cardiac tissue by restoring oxygenated blood flow to the infarcted area, typically by interventional catheterization procedures [117,118]. The sudden re-introduction of oxygen and blood flow to the failing tissues causes an immediate, adverse response in the tissues. This event is characterized by high levels of oxidative stress and mitochondrial re-energization and sudden intracellular calcium overload [119,120,121,122,123]. This traumatic response can subsequently lead to inflammation in the cardiac tissue, in which the innate immune system plays a major role in modulating the different stages of MI [124]. In MI, two opposite but complementary subsets of macrophages—resident cardiac macrophages and infiltrating, monocyte-derived macrophages—play major roles in all stages of MI [125]. These infiltrated macrophages produce inflammatory cytokines and chemokines and contribute largely to the initial ischemic response in the tissue post-reperfusion. The precise regulation of tissue repair is critical to the proper remodeling and functional preservation of cardiac function. Aberrant inflammation and macrophage proliferation post-injury can profoundly affect the retention of cardiac function (Figure 3). Here, the role of different macrophage subtypes in different stages of MI will be explored with emphasis on the importance of managing the post-injury inflammatory response for the better preservation of cardiac function.

#### 3.2.1. Cardioprotective Role of Resident Cardiac Macrophages in MI

Resident cardiac macrophages (RCMs) are crucial for the development and homeostasis of the functioning heart, and they also help to mediate the inflammatory response caused by the non-resident, infiltrating macrophages post-MI. The origins of resident cardiac macrophages (RCMs) were originally hypothesized to be derived from bone marrow progenitor cells. However, recent evidence based on fate mapping and single-cell sequencing techniques suggest that resident tissue macrophages are mainly cells that self-renew and proliferate in their local niche [126,127,128]. Resident Cx3cr1^+^ macrophages are the predominant myeloid cell population in the myocardium, and play a major role in the development and homeostasis of the heart [129]. There are many markers that define different subsets of RCMs, such as CCR2, TIMP4/LYVE1/FOLR2 (termed TLF^+^ RCMs), or CX3CR1^+^, which have different differentiation abilities, tissue localizations, renewal capabilities, unique life cycles, and origins [128,130,131]. For example, the CC chemokine receptor (CCR) 2 is a frequently used marker to identify RCM subtypes and their general functions. CCR2^−^ cells are identified as those derived from the primitive yolk sac or from fetal monocyte progenitors and are important for the development of the myocardium [6,132]. Conversely, CCR2^+^ macrophages are derived from fetal hematopoietic and monocyte progenitors and recruited to the myocardial tissue shortly after birth. Depleting the CCR2^−^ macrophages led to worsened cardiac capabilities post-ischemia/reperfusion [133], because whereas CCR2^+^ macrophages play a pro-inflammatory role, CCR2^−^ macrophages have a distinctively anti-inflammatory role and are necessary for the resolution of local inflammation. CCR2^−^ cells are thus the major RCM subtype that protects the myocardium from inflammation in myocardial injuries like MI [134]. Gene expression profiling suggests that RCMs express a repertoire of distinct, M2-like macrophage markers such as Mrc1, CD163, and Lyve-1 and play a cardioprotective role in the heart [129]. Other RCM sub-classifications and markers have been reviewed [135], and will not be explored in depth in this review [135].

#### 3.2.2. Macrophages Contribute to Acute MI-associated Inflammation

Whereas resident macrophages in the heart tissue are typically thought to exert a cardioprotective role, non-resident infiltrated macrophages play an opposing, pro-inflammatory role. In the immediate post-injury response, RCM macrophages die in the infarcted tissue and increased numbers of immune cells are recruited to the area. In MI, recruited CCR2^+^ macrophages are the cells that are typically thought to exert pro-inflammatory functions [125,134]. Bone-marrow- or spleen-derived CCR2^+^ monocytes respond to chemoattractants and cytokines such as monocyte chemoattractant protein 1 (MCP-1) secreted by CCR2^+^ RCMs [136]. Additionally, the tissue-resident CCR2+ macrophages promote the secretion of MCPs and the mobilization of the circulating monocytes via MYD88-dependent mechanisms [133]. The depletion of CCR2+ cells immediately prior to an ischemia/reperfusion injury model resulted in reduced monocyte recruitment and improved cardiac function. The newly recruited macrophages mediate the inflammatory response by producing cytokines such as MCP-1, TNF-ɑ, IL-1, and IL-6 [136,137,138,139,140,141,142,143,144,145,146,147,148,149,150,151] at a much higher rate than the resident CCR2^+^ macrophages [152]. These cytokines exacerbate tissue inflammation by increasing pro-inflammatory cytokine production in adjacent immune cells and recruiting additional immune cells to the area.

#### 3.2.3. Macrophages in MI-Associated Scar Tissue Formation

The infarcted heart tissue will undergo a period of complex and delicate scar tissue formation and tissue remodeling to salvage heart function. This process is largely mediated by macrophages, which interact with other resident cell types, such as cardiomyocytes, endothelial cells, fibroblasts, and lymphocytes, to trigger the remodeling process. Not only can macrophages facilitate extracellular matrix turnover and cardiac fibroblast activation, but they can also directly contribute collagen to the fibrotic scar as shown in zebrafish heart regeneration and mouse heart repair models [153,154]. It has been shown that macrophages can adopt a fibroblast-like (positive for type I collagen, fibroblast-specific protein-1, fibroblast activation protein, etc.) phenotype post-MI to promote scar formation and healing in MI [155], which emphasizes the plasticity of macrophages and the potential for macrophages to differentiate into the needed cell type upon injury.

### 3.3. Macrophages in Stroke

Stroke is a major class of cardiovascular disease that accounts for 41.1 deaths per 100,000 people, and an estimated 9.4 million Americans 20 years or older self-reported having a stroke [156]. A stroke happens when blood flow is interrupted to any part of the brain, causing widespread neuronal death and neurological damage. There are two major types of strokes: ischemic stroke and hemorrhagic stroke. Ischemic strokes are caused by clots that occlude blood vessels supplying blood to the brain and account for the majority of strokes. Hemorrhagic strokes are caused when weakened blood vessels, such as aneurysms or arteriovenous malformations, rupture, causing bleeds in the brain and disrupting proper blood flow [157]. In a stroke, the damage to the blood–brain barrier (BBB) causes major immune cell recruitment to the infarcted tissue, where they mediate an inflammatory response [158].

Monocytes/macrophages play a dual role post-stroke and are highly implicated in the post-stroke inflammatory response and the preservation of brain function post-stroke. Infiltrating monocytes/macrophages were originally thought to play deleterious roles in strokes and to cause pathological impairment. However, mounting evidence has shown a neuroprotective role of macrophages in the post-stroke response, in which they participate in the functional repair of the CNS [159,160,161,162,163]. This discrepancy is likely due to differences in the local microenvironment, the localization of the cells, and the window of time post-stroke. The in vivo model of stroke, genetic background of animals, and the presence of comorbidities such as infections may also affect the results of each study to account for further observed differences.

#### 3.3.1. Infiltrated Macrophages Are Present in the Post-Stroke Brain

Infiltrating monocytes and macrophages display a distinct functional phenotype and transcriptional profiles in the ischemic area [164,165]. Through deep characterization of tissue-resident leukocytes in the cerebral meninges and parenchyma, Beuker et al. have identified that leukocytes exhibit a site-specific response to strokes. They are characterized by a unique subset of myeloid cells of resident microglial origin and are only present in the brain post-stroke (using the middle cerebral artery occlusion stroke model; MCAO). Blocking markers for this cluster of cells partially attenuated the post-stroke morbidities, suggesting the feasibility of using antibodies against stroke-specific cell markers as a therapeutic [166].

The patrolling murine monocytes that respond to tissue injuries are defined as either pro-inflammatory (Ly6C^hi^CCR2^+^CX3CR1^lo^) or anti-inflammatory (Ly6C^lo^CCR2^−^CX3CR1^hi^). The former subset of monocytes generally has a shorter half-life and is selectively recruited to the area of inflammation [167,168,169]. The anti-inflammatory class generally has a longer half-life and preserves endothelial integrity, which is believed to reduce inflammation [167,170]. Recruited monocytes follow a chemokine gradient to the ischemic cerebral lesion post-stroke, which is largely mediated by inflammatory cytokines including MCP-1 and its receptor CCR2 [171,172,173,174,175,176]. The upregulation of integrins and adhesion molecules such as VCAM-1 in the endothelium also plays a major role in the interaction and subsequent infiltration into the brain by immune cells [177,178,179].

#### 3.3.2. Local Microglial and Macrophage Populations Undergo Reprogramming in Post-Stroke Cerebral Tissue

Macrophage reprogramming and polarization is a major contributor to the exacerbation of the period of inflammation following a stroke. In addition to the recruited monocyte/macrophage populations, it is also critical to account for the participation of local, macrophage-like immune cells called microglia in the post-stroke response, which will be described here. In a model of focal transient cerebral ischemia, macrophages were shown to initially exhibit a “good”, pro-phagocytotic and anti-inflammatory M2-like phenotype. Then, about a week post-injury, these macrophages transitioned to a “sick”, pro-inflammatory M1-like phenotype, which is suggested to be the mediator of the poor longer-term prognosis of post-stroke symptoms [180]. The promotion and preservation of the initial M2-like phenotype is thus believed to be beneficial for post-stroke treatment, and has been the motive behind many studies.

Uncovering the molecular mechanisms and modulators of the M2–M1 macrophage axis will be highly beneficial to translating this phenomenon into a therapeutic treatment. For example, the discovery that the pharmacological activation of M2-polarizing factors such as PPAR*γ* can correct the hyperglycemia-induced pro-inflammatory polarization of monocytes and macrophages in infarcts has led to potential therapeutic target discovery [181,182]. Pioglitazone, oleoylethanolamide, and malibatol A are all PPAR*γ* activators, which have been shown to exert neuroprotective effects by targeting the M2 microglial/monocyte/macrophage polarization process [181,183,184]. Other molecules, either naturally occurring or artificial, have been shown to improve post-stroke outcomes by promoting M2 microglia/macrophage polarization. Berberine, a naturally occurring molecule in the body, promoted M2 microglia and decreased M1 microglia populations via the AMPK pathway in a transient MCAO model [185,186]. Isostevial sodium also promoted M2 polarization in microglial/macrophage populations in both acute and chronic timepoints of a cerebral ischemia model by inhibiting a GAS5/miR-146a-5p/Notch1 signaling pathway [187]. Additional signaling axes that modulate M2 polarization in microglia/macrophages such as the STAT6/Arg1 pathway can also increase the resolution of inflammation, efferocytosis, and improve stroke outcomes [188]. The modulation of the STAT3 pathway may be an additional way to target M2 polarization in stroke [189,190]. MicroRNAs (miRNAs) can modulate gene expression in a post-transcriptional manner by binding to and regulating mRNAs and have been shown to play a role in microglia/macrophage polarization. miR-124 and miR-183 have been shown to shift microglial polarization from an M1 to an M2 state and reduce pro-inflammatory cytokine secretion via the NF*κ*B pathway, respectively [191,192]. Minocycline and metformin have also been shown to enhance levels of M2 polarization by targeting the NF*κ*B pathway in strokes [193,194,195].

From a converse perspective, inhibiting the polarization of microglial/macrophage towards an M1-like state is an alternative approach to targeting macrophage polarization in stroke. For example, modulating the CD8 signaling pathway, which independently promoted M1 microglia/macrophage polarization in a rat model of cerebral ischemia via M1 stimulatory pathways through signal transducers such as Syk, presents therapeutic value [196]. Using compounds like glycine or baicalin to inhibit M1 polarization via the modulation of the NF*κ*B pathway is another possible method for the attenuation of stroke-associated inflammation [197,198]. miR-155, an established modulator of microglial phenotypes, has been shown to promote M1 polarization post-stroke, and its inhibition significantly alters the time course of inflammation, changes cytokine production, and promotes functional recovery after experimental stroke [199]. In short, the modulation of microglia/macrophage polarization towards an anti-inflammatory and against a pro-inflammatory M1 phenotype can play pivotal role in determining post-stroke outcomes and shows incredible translational potential.

### 3.4. Macrophages in Other Cardiovascular Diseases

#### 3.4.1. Cardiac Arrythmias

Cardiac arrythmias are defined as a disrupted heart rhythm. The more fatal arrythmias occur in the ventricles and are often acutely triggered by MI or chronically by improper scar formation within the myocardium. Cardiac excitation–contraction coupling is mediated by cardiomyocytes, which coordinate this process among all cardiomyocytes via intercellular-signaling gap junctions such as connexin 43 (Cx43) [200]. Disruptions in intercellular signaling and Cx43 function or Cx43 levels can lead to cardiac arrythmias. Interestingly, RCMs play a cardioprotective role in arrythmias via direct or indirect mechanisms.

RCMs are highly implicated in the electrical conduction and regulation of cardiomyocytes, with which they are physically and functionally coupled to assist normal AV node conduction and the modulation of the electrical activity and membrane of cardiomyocytes [201]. For example, RCMs can produce growth factors such as amphiregulin, which is a key regulator in the phosphorylation and translocation of Cx43 in cardiomyocytes, functionally linking cardiomyocytes and RCMs [201,202]. RCMs also help maintain cardiac electrical conduction through the regulation of gap junction formation. Sequencing analyses of mononuclear cells from patients with post-MI arrythmias also showed an increase in macrophage polarization from an M0- to an M1-like inflammatory subset compared to post-MI patients without arrythmias. The increased arrythmias were linked to the upregulation of the Kcnn4 gene and the activation of the associated KCa3.1 ion channel [203]. The identification of other ion channels and the characterization of the passive and active electrophysiological activities of murine RCMs have unveiled multiple voltage-gated and outward- and inward-rectifying potassium channels (Kv1.3, Kv1.5, and Kir2.1) in the regulation of cardiac macrophage currents [204], the mediation of proper electrical signaling, and arrythmia. Such work has led to the generation of a computational model to describe cardiac macrophage electrophysiology and their contributions to cardiomyocyte action potentials [204]. Such models can then be used to investigate the effect of dysfunctional ion channel signaling in pathophysiological remodeling in diseases like arrythmia.

Cardiac arrythmia often happens post-MI due to the buildup of dying or dead cardiomyocytes, which can lead to the functional heterogeneity of signal transduction. The depletion of macrophages impaired the clearance of such cellular debris in vivo and decreased the expression of MerTK, a major receptor in macrophages responsible for the mediation of efferocytosis and dying cell clearance, which triggers post-MI ventricular arrythmias [205]. Additionally, the macrophage-mediated removal of expelled mitochondria from stressed cardiomyocytes post-MI promoted a steady state of cardiomyocyte metabolism and cardiac function. When the macrophages were depleted, the mitochondrial morphology in cardiomyocytes was highly abnormal and mitochondrial function was greatly compromised, contributing to a vicious cycle of dysfunctional cardiomyocyte function and arrythmia formation [205]. In the case of atrial fibrillation (AFib), the most common type of arrythmia, the recruitment of a population of CCR2^+^ macrophages is a defining feature of this disorder. The inhibition of macrophage migration to the myocardium using CCR2^−/−^ mice reduced arrythmias in a hypertensive, obese, mitral-valve regurgitation (HOMER) mouse model by inhibiting SPP1, a pleiotropic signal involved in the crosstalk with local cells [206].

Uncovering the role of macrophages in arrythmias has revealed these cells as a possible new target for arrythmia management and treatment in patients. The association of macrophages with both arrythmias and arrythmia-triggering CVDs such as MI shows that targeting macrophage function or macrophage-secreted compounds may exert more cardioprotective effects. However, the complexity and heterogeneity of macrophage populations pose a problem when targeting these cells, but identifying recruited macrophages as a major adverse regulator of many myocardial diseases suggests a specific population of cells with targeting potential.

#### 3.4.2. Hypertension

Hypertension is the leading, most modifiable risk factor for CVDs including ischemic heart disease and stroke and is the underlying cause of about 7 million deaths annually [207,208]. The prevalence of hypertension, commonly referred to as high blood pressure, has increased in the past four decades. It is especially prevalent in low- or middle-income countries due to less awareness, lower rates of treatment, and less management of care. Despite treatment with anti-hypertensive agents, there is still an insufficient amount of control over blood pressure, which suggests that novel therapeutic approaches are necessary to better manage this disease and its associated risks. Hypertension largely exerts its effect by damaging the integrity of the arterial lining and has also been shown to play a major role in chronic kidney disease and end-stage renal disease [209,210,211].

Hypertension is associated with the overactivation or dysfunction of the renin–angiotensin system (RAS). Recent studies have highlighted that angiotensin-II (AngII)-induced hypertension is associated with chronic, low-grade renal and vascular inflammation caused by immune system dysfunction [212,213]. In an AngII hypertension model, monocyte/macrophage populations appeared to have a major role in regulating renal and vascular inflammation in the renal and arterial tissues. Many studies characterizing the cytokine landscape of hypertensive patients have shown elevated levels of inflammatory cytokines like IL-6, IL-1β, IL-1ɑ, IL-18, IL-2, IL-8, TNF-ɑ, IFN-*γ*, C-reactive protein, and MCP-1 in the serum [214]. MCP-1 is highly expressed in the kidney and in the vasculature in hypertension [215], which elevates the recruitment levels of CCR2^+^ monocytes to the area [216], and the depletion of MCP-1, CCR2, and/or monocytes decreases myeloid cell infiltration into the renal and vascular systems or the chronic hypertensive response to AngII [216,217,218]. In a clinical, post-mortem study on hypertensive kidneys, the density of CD68^+^ monocyte/macrophages was significantly upregulated compared to normotensive kidneys, further suggesting a role for monocyte/macrophage infiltration and expansion in hypertensive renal tissue [219].

In response to increased sympathetic outflow modulated by the central nervous system, elevated levels of chemoattractants like MCP-1 in the kidneys recruits and promotes the infiltration of macrophages into the renal medulla. The incubation of a human monocyte cell line with AngII has also been shown to increase NF*κ*B activation and pro-inflammatory cytokine production (i.e., TNF) in an angiotensin-II-type-1-receptor (AT_1_)-dependent manner [220]. The disruption of other macrophage-associated cytokines or their receptors, such as IL-1/IL-1R, has also been shown to limit blood pressure elevations in various ways, including the modulation of sodium reabsorption in the nephron [221]. Additionally, the ablation of monocytes/macrophages also reduced reactive oxygen species (ROS) and superoxide production in aortic tissues, which ameliorated vascular dysfunction associated with AngII-induced hypertension [217].

The polarization of macrophages in hypertension is undoubtedly important in both the acute and chronic timepoints of the AngII model. Aorta-infiltrated macrophages were present by day 7 in an AngII model, and they initially exhibited a pro-inflammatory M1-like phenotype, yet CD206^+^Arg1^+^ M2-like macrophages were more prevalent at day 14 and associated with fibrosis, elastin loss, and hypertension [218,222]. It is unclear if the M1-like macrophages transition into the M2-like state or if they are replaced by additional infiltrating M2-like monocyte/macrophages. The increased presence of M2-like macrophage populations in later timepoints of the hypertension model challenges the idea that M2 macrophages are beneficial in all clinical diseases, as the accumulation of these M2-like macrophages appears to contribute to the poor prognosis of hypertension. Thus, it appears to be more promising to target the initial monocyte recruitment to the tissues through chemoattractant receptors like CCR2 rather than manipulate macrophage polarization states within the tissues post-infiltration.

## 4. Conclusions

When developing therapeutic approaches targeting macrophages for CVDs or inflammatory diseases, it is important to recognize that macrophages have different regulatory axes in different tissues, conditions, and diseases. In other words, the role of the macrophage is highly diverse, and the targeting of macrophages as a therapeutic target must be spatially and temporally specific. While in pursuit of a macrophage-specific treatment for cardiovascular diseases, one must consider potential comorbidities that influence the development of cardiovascular disease. For example, macrophages are a central modulator of rheumatoid arthritis, a disease that results in a heightened disposition to atherosclerosis [223]. Thus, in vivo models with other pathological comorbidities in addition to CVD are valuable models to assess the pleiotropic nature of macrophage function in the entirety of the in vivo system.

Several key processes involving macrophages appear to be common factors among the most prevalent and the most fatal CVDs. Dysfunction in macrophage-mediated activities, such as macrophage polarization, cytokine secretion leading to a vicious inflammatory response with excessive, prolonged immune cell recruitment, and impaired efferocytosis, are shared factors that can significantly influence disease prognosis, either improving or worsening the condition. Understanding the molecular mechanisms of all these processes and revealing a comprehensive list of regulatory mechanisms will be extremely beneficial to finding therapies and management methods for a diverse array of inflammatory diseases in addition to CVDs. Identifying other proteins, ion channels, and intermediate factors in macrophage-specific inflammatory, phagocytic, or polarization pathways is crucial for the successful translation of macrophage research from the bench to bedside.

With many similarities between murine and human macrophage behavior and the ability to isolate and manipulate these cells both in vivo and in vitro, it is important for future research to focus on the macrophage-specific, tissue-specific modulation of macrophage polarization or inflammatory status in cardiovascular diseases and other diseases. With the advancement of drug delivery methods (including antibodies, nanoparticles, viral vectors, small-molecule agonists and antagonists, etc.), the ability to target macrophages in a specific manner becomes more feasible [224]. Moreover, advances in macrophage phenotyping techniques will allow for the better targeting of specific macrophage subtypes specific to each disease to avoid undesirable off-target effects. The prevalence of macrophages in chronic and acute inflammatory diseases makes it necessary to understand their role in a variety of diseases, including cardiovascular diseases. As cardiovascular disease is the major driver of the majority of deaths worldwide, uncovering novel therapeutic targets from an immunomodulatory perspective holds exciting promise for the development of more effective therapies.
